# Association of Short-Term Changes in Menstrual Frequency, Medication Use, Weight and Exercise on Bone Mineral Density in College-Aged Women

**DOI:** 10.3390/ijerph191610363

**Published:** 2022-08-19

**Authors:** Stacie H. Fleischer, Annalisa K. Freire, Katie Brown, Andrew Creer, Dennis L. Eggett, Susan Fullmer

**Affiliations:** 1 Department of Nutrition, Dietetics and Food Science, College of Life Sciences, Brigham Young University, Provo, UT 84602, USA; 2 Department of Nutrition, Dietetics & Food Sciences, College of Agriculture and Applied Sciences, Utah State University, Logan, UT 84322, USA; 3 Department of Statistics, College of Physical and Mathematical Sciences, Brigham Young University, Provo, UT 84602, USA; 4 Department of Exercise Science & Outdoor Recreation, College of Science, Utah Valley University, Orem, UT 84058, USA

**Keywords:** bone mineral density, BMD, oral contraceptives, amenorrhea, weight loss, calorie restriction, young adult, women, visceral adipose tissue

## Abstract

To evaluate if experiencing a short-term exposure (18-months) to factors such as menstrual irregularities, dieting, changes in exercise or body weight, and medication usage is associated with bone mineral density (BMD) in college-aged females. A retrospective survey assessing health behaviors during a recent 18-month time period and a DXA scan were completed in 641 females. A total of 45.5% of participants reported amenorrhea during the 18-month time period. Those who experienced amenorrhea had lower femoral neck BMD (*p* = 0.018), trochanter (*p* = 0.018) and spine BMD (*p* = 0.022) compared to eumenorrheic women. Lifetime oral contraceptive usage longer than six months was negatively associated with BMD at femoral neck (*p* = 0.018) and total hip (*p* = 0.021). Women who lost weight trended towards having the lowest BMD at all sites compared to women who gained weight. Following a very-low calorie diet during the time period was negatively correlated with spine BMD (*p* = 0.001). Time spent in vigorous and very vigorous activity was weakly correlated with some hip BMD sites but time spent in extremely vigorous activity was not. In conclusion, females who experienced weight loss, amenorrhea, or a very low-calorie diet within an 18-month period of time in young adulthood had lower BMD. Additionally, oral contraceptive usage for longer than six months during their lifetime was associated with lower BMD.

## 1. Introduction

Bone mineral density (BMD) (g/cm^2^) is currently the primary measure of bone health. Accrual of BMD occurs rapidly from infancy through adolescence [1]. During young adulthood, BMD peaks during the third decade of life, followed by a slow decline but then is accelerated after menopause [1]. While peak bone mass is primarily determined by genetics [2], exposure to various important lifestyle factors during adolescence and young adulthood could affect total bone mass accrual and therefore, may influence the risk of osteoporosis later in life [3].

Research has consistently identified biological and lifestyle factors that can affect BMD. Biological factors include age [4,5], age at menarche [6], menstruation/ovulation [7,8], estrogen levels [9]) and secondary amenorrhea [7,8,10]. Lifestyle factors include body weight [11], lean body mass [4,12], weight loss [13,14], dietary factors such as energy availability, calcium, and vitamin D [1,15], physical activity [1], load-bearing exercise [16]), smoking, alcohol intake and certain medications [2,17,18,19]. 

As peak bone mass accrual typically occurs between ages of 18–25 [2], studying factors associated with bone health during young adulthood is warranted and much of the existing research in this population is among female athletes. Low energy availability [7,15], eating disorders [20,21,22], secondary amenorrhea [7] and resultant low estrogen [9], lack of weight-bearing exercise [23], low body weight [24], and some medications [17,25] are negatively associated with BMD in these populations. Secondary amenorrhea is prevalent among other groups of young adult women including international volunteers (~30%) [26,27], and college students (~21%) [27].

However, short term (e.g., 18-months) lifestyle changes that may occur during young adulthood may also be associated with BMD. During young adulthood, women often transition to independent living including cooking for themselves, attending college/university, international volunteer service, etc. This study aims to evaluate the BMD of a large population of healthy young adult women who experience shorter term exposure (i.e., 18 months–approximately the first 1.5 years of college or the approximate duration of international volunteer service) and assess associations with common risk factors experienced in young adulthood. By identifying modifiable risk factors associated with lower BMD, young adult women could consider behavior changes that might impact their bone health.

## 2. Materials and Methods

### 2.1. Design and Study Population

A large retrospective, observational survey was completed by young adult women. Several other studies have used a similar approach to assess the association of lifestyle factors to current BMD [28,29,30]. Details of the survey development and design are reported elsewhere [27]. In brief, an online survey using Qualtrics software (Qualtrics Version March 2019, Provo, UT, USA) was developed and refined. The survey was sent to eligible subjects from email lists provided by six universities in the western United States. Email lists included currently enrolled female students ages 21–26 years (born between 1993–1998). The study was conducted in accordance with the Declaration of Helsinki, and the protocol was approved on 13 March 2019 by the primary sponsoring institution (Brigham Young University) (approval code: F18518). The institutional review boards at each participating university also approved the study. All subjects gave their informed consent for inclusion before they participated in the study. Survey participants were also invited to have a DXA scan to measure body composition and bone density.

### 2.2. Survey

In the survey subjects were asked to provide retrospective information related to an 18-month period of time on the following: physical activity, menstrual history, weight history, medications (steroids, antacids, Proton pump inhibitors (PPIs), selective serotonin reuptake inhibitors (SSRIs), oral contraceptives (OC), chemotherapy agents, anticonvulsants and methotrexate) and medical diagnoses that are known to affect BMD. Questions regarding overall changes in physical activity patterns and levels of exercises were based on metabolic equivalents (METs). The categories of MET activities were divided into moderate (3–5.9 METs) and vigorous (6–10.9 METs) [31]. Vigorous intensity exercise was further categorized as: very vigorous (11–14.9 METs), and extremely vigorous (15–18 METs). In an effort to reduce respondent burden, activities that were light or very light were not included in the survey. Subjects also described changes in their physical activity prior to the study period compared to the 18-month study period in terms of exercising more, less, or no change.

Oligomenorrhea was defined as nine cycles or less in one year and amenorrhea as four cycles or less in one year [32]. Weight history was assessed by subjects reporting their highest and lowest body weight, as well as overall weight trend (gain, loss, gain and loss, maintenance) during the study period. Subjects reported any medications influencing BMD that they were currently taking or had taken for six months or longer.

### 2.3. DXA Scan

Subjects were invited to have a DXA scan to measure body composition and bone mineral density. An iDXA (GE Healthcare, Lunar Prodigy; Chicago, IL, USA) located at only one institution limited the total number of survey participants who had a scan. Each participant was required to complete a urinary pregnancy test immediately prior to the DXA scan. Eligibility for receiving a DXA scan included a negative pregnancy test result, absence of metal implants, and no magnetic resonance imaging or contrast agents within the previous five days. A form was provided that detailed the procedures and risks of the DXA scan and written informed consent was obtained from each subject. Members of the research team were trained in operating the DXA machine and performing urinary pregnancy tests. BMD measurements were taken of whole body, lumbar spine and both hips as well as body composition (total mass, fat free mass, lean mass, fat mass, and visceral adipose tissue (VAT)) measurement for each participant. 

### 2.4. Statistical Analysis 

Statistical Analysis System (SAS version 9.4; Cary, NC, USA) was used for all statistical analyses. Descriptive statistics including mean, standard deviation and range were used to summarize continuous variables. Frequencies and percent of total were calculated to summarize categorical data. Associations between continuous and categorical data and single factors of interest were assessed using analysis of variance (ANOVA) and chi-squared distribution. Linear regression was used to assess covariates of menstrual and bone health. ANOVA and Tukey-Kramer were used to compare differences between groups. To reduce the risk of Type 1 and Type 2 errors, levels of significance were adjusted based on the number of multiple comparisons as follows: for <5 comparisons, *p*-value < 0.05; between 5–20 comparisons, *p* < 0.0125; for 21–40 comparisons *p* < 0.005; and for more than 40 comparisons, *p* < 0.001.

## 3. Results

### 3.1. Subject Characteristics

A total of 4015 participants took the survey, of which 324 were excluded for incomplete data. Of the remaining 3691 participants who completed the survey, 641 subjects had also completed a DXA scan. All but eight subjects were Caucasian (mean age 22.9 years); three subjects were Hispanic (mean age 22.3 years) and four subjects were of Asian descent (mean age 24.1 years). Mean body weight of subjects was normal to overweight. Subjects’ descriptive characteristics are presented in Table 1.

### 3.2. Menstruation

**Figure 1:**
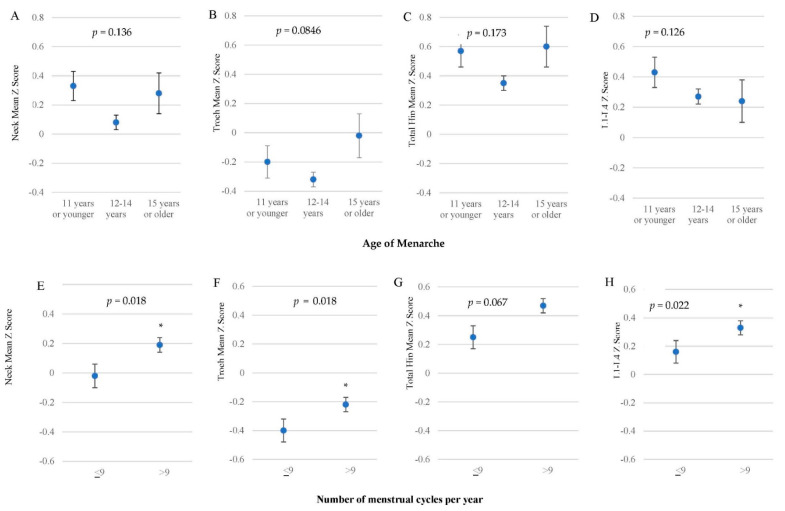
Comparison of age at menarche and number of menstrual cycles per year during 18-month study period of interest with mean ± standard error BMD Z-score at each measurement site. (**A**–**D**), Age of menarche: 11 years or younger (*n =* 84); 12–14 years (*n =* 368); 15 years or older (*n =* 48). No significant difference between groups; *p* < 0.0125. (**E**–**H**), Number of menstrual cycles per year: ≤9 (*n =* 230); >9 (*n =* 276), * indicates a significant difference, *p* < 0.05. Survey respondents were not required to answer every question, so n might not total 641.

Secondary amenorrhea was reported in 45.5% of subjects during the 18-month period. The majority of participants (72.6%) reported age of menarche between 12–14 years, 16.6% reported menarche before 11 years old and only 9.5% reported menarche after the age of 15. Age of menarche was not associated with BMD in this cohort. Women who reported secondary amenorrhea (≤9 cycles per year) during the 18-month period had significantly lower BMD in three of the four measured bone sites. Figure 1 shows age of menarche and number of menstrual cycles per year and corresponding mean BMD Z-scores at each measurement site.

### 3.3. Medications

**Figure 2:**
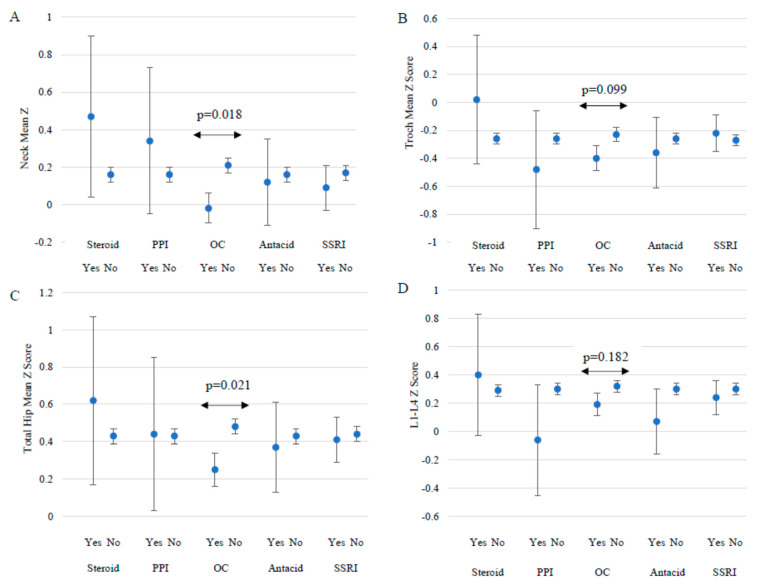
Lifetime medication usage and mean ± standard error BMD Z-score at each measurement site. (**A**–**D**), PPI = proton pump inhibitors. OC = oral contraceptives. SSRI = selective serotonin reuptake inhibitors. Steroids/prednisone yes (*n =* 5), no (*n =* 626); Proton pump inhibitors yes (*n =* 6), no (*n =* 625); Oral contraceptives yes (*n =* 128), no (*n =* 503); Antacids/aluminum yes (*n =* 17), no (*n =* 614); Selective serotonin reuptake inhibitors yes (*n =* 65), no (*n =* 566).

The most common medications among all subjects were oral contraceptives (20.3%), serotonin-reuptake inhibitor medications (10.3%), antacids (2.7%), proton pump inhibitors (0.9%), and prednisone or steroids (0.8%). Use of chemotherapy agents, anticonvulsants, and methotrexate was reported by only one participant each, and no participants reported usage of anticoagulants; therefore, these medications were not included in analysis of BMD. In women who reported oral contraceptive use for longer than six months in their lifetime, BMD Z-scores trended lower at all measurement sites compared to those who did not report oral contraceptive usage (Figure 2) (*p* = 0.018). There was no significant relationship with steroid usage (*p* = 0.47), PPIs (*p* = 0.66), or Antacids (*p* = 0.43), or SSRIs (*p* = 0.63).

There is no significant difference for usage (yes) vs. no usage (no), *p* < 0.0125. Survey respondents were not required to answer every question, so n might not total 641.

### 3.4. Weight Change and Dieting

**Figure 3:**
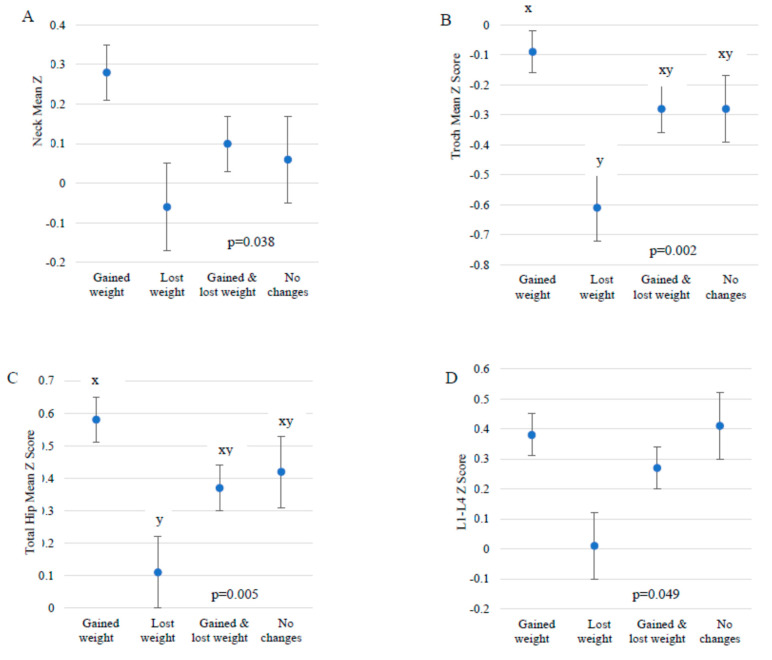
Weight change trend during 18-month study period of interest and mean ± standard error BMD at each measurement site. (**A**–**D**), Gained weight *n =* 176; Lost weight *n =* 80; Gained and lost weight *n =* 172; No changes *n =* 79. Columns with different XY indicate *p* < 0.0125 (Tukey-Kramer).

Eighty-four percent of subjects recorded some form of weight change during the 18-month study period. The BMD Z-scores of those who lost weight trended lower at all measurement sites. compared to those who gained weight, lost and gained weight, or had no weight change (Figure 3). Similarly, those who gained weight over the 18-months tended to have higher BMD Z-scores at all measurement sites, but the differences were only significant in the trochanter and total hip when weight gain was compared to weight loss. The most common methods of weight loss for all subjects were a very-low calorie diet, high protein/low carbohydrate diet, additional exercise, and fasting for non-religious purposes. Women who had engaged in a very low-calorie diet during the 18-month study period had significantly lower spine BMD Z-scores (Table 2). 

### 3.5. Physical Activity (PA)

Most participants (80.9%) reported change in physical activity levels during the 18-month study period of interest. While there were statistically significant differences in BMD scores based on the PA changes, the only significant differences between groups was when comparing “exercising less” and “no changes” for the hip measurement sites. However, for the BMD Z-scores, women who exercised less trended towards higher BMD Z-score at the neck, trochanter, and total hip compared to those who exercised more (Table 3). Correlations between BMD and time spent in the various categories of MET hours were mostly small except for extremely vigorous activity which had higher but no significant correlations at *p* < 0.0125. Only vigorous and very vigorous were significantly correlated to the trochanter BMD Z-scores. (Table 4).

### 3.6. Body Composition

Table 4 shows the correlations between body composition and BMD Z-scores. Total bone mass and lean mass were significantly related to all measured bone sites; fat mass was significantly related to all hip sites. Visceral adipose tissue (VAT) correlated with total hip BMD Z-scores but not with other measured sites. 

## 4. Discussion

The primary aim of this study was to evaluate whether temporary changes in menstrual patterns, medication usage, weight history, and exercise was associated with BMD-Z scores in a large sample of college-aged women.

There were no significant differences in BMD Z-scores between ages at menarche. An early age at menarche can indicate earlier exposure to estrogen which could result in higher BMD [33]; and an older age at menarche has been associated with lower BMD in older age [34]; however, we did not see a relationship between age of menarche and BMD in our study cohort, which is consistent with other studies [35,36]. However, more frequent menstrual cycles (>9/year) were associated with higher BMD Z-scores at all sites except at the total hip. Several studies have demonstrated that when oligomenorrhea or secondary amenorrhea are due to eating disorder or excessive exercise there is an associated lower bone BMD [37,38,39,40].

Overall, 35.8% of subjects reported current or lifetime usage of common medications that might affect bone density. Just over 20% of participants took oral contraceptives for at least six months over their lifetime; while there might be a modest trend of lower BMD in our study population, only neck and total hip BMD Z-scores approached significance. The potential detrimental effects of oral contraceptives in young adults women has been previously demonstrated [18,19]. Almstedt et al. [17], found that college-aged oral contraceptive users had elevated bone turnover, declines in spine BMD, and lack of bone acquisition of the whole body over 12 months compared to their non-oral contraceptive user counterparts. However, several studies have explored the protective effect of oral contraceptives on BMD in young women with anorexia nervosa. These participants had lower BMD compared to controls but had higher BMD than other anorexia nervosa patients who were not taking oral contraceptives [41,42]. Other studies have suggested that the amount of ethinyl estradiol may alter the consequence on BMD [43]. Very low doses of estrogen were associated with lower BMD compared to low doses or no supplementation [17,44] Thus, the effect of oral contraceptives on BMD may be influenced by one’s eating disorder status and dose of ethinyl estradiol. However, due to the low incidence of eating disorders in our study population, the negative effects of oral contraceptives on BMD in a non-anorexic population is consistent with the literature. Steroid, PPI, and antacid use in our population was very small which made it difficult to detect an association, if one exists. However, 65 subjects had used SSRIs but there was no association with BMD Z-scores. The role of SSRIs on BMD is not clear [45].

Women who lost weight during the time period of interest had significantly lower trochanter and total hip BMD than those who maintained weight, but there was a consistent trend of lower BMD Z-score in all measured sites compared to the other categories of weight change (Figure 3). In addition, there was a consistent trend of higher BMD Z-scores at all measured sites compared to women who gained. It is well known that weight loss is often associated with a lowering of BMD and a higher body weight is correlated to higher BMD [46,47]. Study participants who followed a very low-calorie diet during the 18 months of interest had significantly lower spine BMD than those who used other weight loss methods or no weight loss method. Other studies have demonstrated that exercised-induced weight loss results in increased or maintained BMD compared to calorie-restriction induced weight loss [17,46,48]. 

We inquired about changes in physical activity with two approaches. First, we asked subjects to recall if their overall physical activity changed (either exercised more, less, no change or didn’t know) during the 18-month period. The women who reported to have exercised less had significantly higher BMD Z-scores at all hip sites compared to those who reported no change but there were no difference when compared to having exercised more or didn’t know. While not statistically significant, it is notable that those who exercised less had the highest BMD Z-scores at all bone sites. The second approach to assess physical activity was to ask subjects to estimate how much time they spent weekly in moderate (3–5.9 METS), vigorous (6–10.9 METS), very vigorous (11–14.9 METS) or extremely vigorous activity (15–18 METS). The mean hours/week subjects spent in the various categories were 11.3 h/week for moderate intensity activity, 3.4 h/week of vigorous intensity activity, one hour/week of very vigorous activity and only 0.3 h/week, respectively. Correlations of METS categories to BMD were modest and only two sites reached statistical significance (*p* < 0.0125). While moderate intensity exercise did not correlate with BMD, both vigorous and very vigorous activities did. However, it is likely very difficult to accurately recall the amount of time spent in various activities. Interestingly, extremely vigorous activity had no relationship to BMD and none of the activity intensities impacted lumbar spine. Research on the impact of aerobic exercise on lumbar spine BMD are mixed. Liang, et al. [49] found that high impact step aerobics resulted in an increase of heel BMD, but not at any hip or lumbar spine site. Fullmer [50] did not find a relationship to time spent in aerobic exercise on lumbar spine BMD in a healthy young adult group of women; while Burrows et al. [51] found that distance run per week was negatively associated with lumbar spine and femoral neck BMD in recreational and elite female runners. None of the MET activity levels in our study were negatively correlated with BMD.

Bone mass, lean mass and fat free mass were positively correlated with BMD, and total fat free had the strongest correlations to all BMD sites; however, the effect on lumbar spine was low. This is consistent with similar studies comparing body composition and bone density in young adult women where lean mass was the strongest predictor of BMD. Regarding adipose tissue, total fat mass was a weak predictor of BMD at all measurement sites, which is consistent with various studies where there was a weak or inverse correlation with BMD [52,53,54]. Marchand et al. [55] found that increased adipose tissue was linked to an increase of circulating estrogen levels, which may explain some impact on BMD. VAT was the lowest adipose predictor of BMD Z-scores and was only associated with the total hip BMD. 

Strengths of this study include a large sample size of young adult females; we used the same DXA scanner for all the bone density scans and trained personnel conducted all of the measurements. The results of our study are consistent with the findings of other published findings respecting the association of oral contraceptive usage, weight loss, and restrictive eating and BMD in young adult women.

There are some limitations to our study. The study was retrospective and relied on subject recall and estimations. The current study did not include all factors that are related to BMD (diet, microbiome, genetics, etc.). For example, consumption of many dietary components such as calcium, vitamin D, energy intake, etc. and an overall balanced diet rich in low fat dairy products, and fruits and vegetables can play a role in BMD [2]. The role of calcium and vitamin D are important modifiable factors in reaching peak bone mass [2]. Other limitations are that the majority of subjects were predominately Caucasian of European ancestry, limiting generalizability to other populations. Additionally, BMD was only measured after the time period of interest, and it is not known if the BMD decreased during the 18-month time period but later increased.

## 5. Conclusions

In summary, lifestyle changes over an 18-month period of time in early adulthood including secondary amenorrhea, weight loss, very low-calorie dieting, and oral contraceptive use longer than six months were associated with lower BMD Z-scores in a large sample of young adult women. Vigorous and very vigorous intensity exercise were modestly correlated with BMD at the trochanter; however exercising less was also correlated with higher BMD Z-scores. Bone mass, lean mass and fat mass were positively correlated with BMD. Future research should include longitudinal studies to better understand how much modified lifestyle factors affect young adult peak BMD and whether the changes are reversible if the lifestyle factor is reversed.

## Figures and Tables

**Table 1:** Descriptive characteristics (age, height, weight, BMI, and BMD measured in (g/cm^2^) and BMD z-scores of study population. (N = 634).

	Mean ± SD	Range
Age	23.3 ± 1.3	21.1–27.2
Current Height (cm)	166.3 ± 7.1	147.3–193.0
Current Weight (kg)	65.5 ± 13.5	42.7–136.4
Current BMI (kg/m^2^)	23.7 ± 4.5	15.8–45.6
**Measurement site**	**Mean ± SD**	**Range**
L_1_–L_4_ BMD (g/cm^2^)	1.22 ± 0.13	0.92–1.71
L_1_–L_4_ Z-Score	0.29 ± 0.96	−1.88–4.16
Neck Mean BMD (g/cm^2^)	1.07 ± 0.14	0.75–2.02
Neck Mean Z-Score	0.16 ± 0.96	−1.97–3.26
Troch Mean BMD (g/cm^2^)	0.83 ± 0.12	0.53–1.37
Troch Mean Z-Score	−0.26 ± 1.02	−2.86–4.52
Total Hip BMD (g/cm^2^)	1.22 ± 0.10	0.75–1.48
Total Hip Mean Z-Score	0.43 ± 0.99	−2.02–3.45

Mean measurement sites indicate average between left and right hip scans.

**Table 2:** Difference of BMD Z-score at each measurement site between weight change patterns and weight loss methods (Mixed Model ANOVA) (N = 632).

Weight Loss Methods	N (%)	Neck Z-Score	*p*-Value	Troch Z-Score	*p*-Value	Total Hip Z-Score	*p*-Value	L1-L4 Z-Score	*p*-Value
		LSM ± SE *		LSM ± SE		LSM ± SE		LSM ± SE	
Liquid diet supplements									
Yes	20 (3.2)	0.36 ± 0.21	0.3563	−0.11 ± 0.23	0.4816	0.62 ± 0.22	0.3838	0.38 ± 0.21	0.6529
No	612 (96.8)	0.16 ± 0.04		−0.27 ± 0.04		0.43 ± 0.04		0.28 ± 0.04	
Very low calorie-diet									
(<1000 kcal)									
Yes	80 (12.8)	−0.04 ± 0.11	0.0417	−0.50 ± 0.11	0.0249	0.15 ± 0.11	0.0073	−0.03 ± 0.11	0.001 **
No	552 (87.2)	0.19 ± 0.04		−0.23 ± 0.04		0.47 ± 0.04		0.33 ± 0.04	
Fasting									
(for nonreligious reasons)									
Yes	32 (5.1)	0.22 ± 0.17	0.6922	−0.19 ± 0.18	0.6888	0.46 ± 0.18	0.8734	0.15 ± 0.17	0.4101
No	600 (94.9)	0.16 ± 0.04		−0.27 ± 0.04		0.43 ± 0.04		0.29 ± 0.04	
High protein/low									
carbohydrate diet									
Yes	46 (7.3)	0.16 ± 0.14	0.998	−0.25 ± 0.15	0.9273	0.40 ± 0.15	0.8501	0.35 ± 0.14	0.6485
No	586 (92.7)	0.16 ± 0.04		−0.26 ± 0.04		0.43 ± 0.04		0.28 ± 0.04	
Additional exercise									
beyond regular training									
Yes	79 (12.6)	0.07 ± 0.11	0.3671	−0.24 ± 0.12	0 .7974	0.35 ± 0.11	0.4184	0.09 ± 0.11	0.0497
No	553 (87.4)	0.17 ± 0.04		−0.27 ± 0.04		0.44 ± 0.04		0.31 ± 0.04	
Diet pills or fat									
burning supplements									
Yes	15 (2.4)	0.29 ± 0.25	0.5988	0.18 ± 0.26	0.0861	0.71 ± 0.25	0.27	0.28 ± 0.24	0.9797
No	617 (97.6)	0.16 ± 0.04		−0.27 ± 0.04		0.42 ± 0.04		0.29 ± 0.04	
Self-induced vomiting									
Yes	15 (2.4)	−0.14 ± 0.25	0.2129	−0.67 ± 0.26	0.1191	0.08 ± 0.26	0.1627	0.03 ± 0.25	0.295
No	617 (97.6)	0.17 ± 0.04		−0.25 ± 0.04		0.44 ± 0.04		0.29 ± 0.04	
Laxative use									
Yes	12 (1.9)	−0.12 ± 0.28	0.3021	−0.41 ± 0.30	0.617	0.12 ± 0.29	0.2699	0.01 ± 0.28	0.32
No	620 (98.1)	0.17 ± 0.04		−0.26 ± 0.04		0.44 ± 0.04		0.29 ± 0.04	
Other									
Yes	44 (7.0)	0.06 ± 0.14	0.4789	−0.44 ± 0.15	0.2322	0.33 ± 0.15	0.4812	0.20 ± 0.14	0.5172
No	588 (93.0)	0.17 ± 0.04		−0.25 ± 0.04		0.44 ± 0.04		0.29 ± 0.04	
None of the above									
Yes	283 (45.2)	0.14 ± 0.06	0.5273	−0.24 ± 0.06	0.564	0.45 ± 0.06	0.7441	0.38 ± 0.06	0.0281
No	349 (54.8)	0.18 ± 0.05		−0.28 ± 0.05		0.42 ± 0.05		0.21 ± 0.05	

All weight history and weight loss results are self-reported historical data of the 18-month study period of interest.* LSM ± SE = Least Square Means ± Standard Error. ** Indicates *p* < 0.001 between yes and no. Survey respondents were not required to answer every question, so n might not total 641.

**Table 3:** Difference of BMD Z-score at each measurement site among physical activity changes (Mixed Model ANOVA) (N = 513).

Physical Activity Changes	n (%)	Neck Z-Score	*p*-Value	Troch Z-Score	*p*-Value	Total Hip Z-Score	*p*-Value	L1-L4 Z-Score	*p*-Value
		LSM ± SE *				LSM ± SE *		LSM ± SE *	
Exercised more	202 (39.4)	0.06 ± 0.07 ^xy^	0.0003	−0.39 ± 0.07 ^xy^	0.0003	0.33 ± 0.07 ^xy^	0.0005	0.23 ± 0.07	0.0165
Exercised less	213 (41.5)	0.33 ± 0.06 ^x^		−0.05 ± 0.07 ^x^		0.61 ± 0.07 ^x^		0.46 ± 0.07	
No Change	92 (17.9)	−0.15 ± 0.10 ^y^		−0.52 ± 0.10 ^y^		0.13 ± 0.10 ^y^		0.08 ± 0.10	
Don’t know	6 (1.2)	0.15 ± 0.41 ^xy^		−0.13 ± 0.44 ^xy^		0.45 ± 0.43 ^xy^		−0.19 ± 0.43	

All physical activity results are self-reported historical data of the 18-month study period of interest. * LSM ± SE = Least Square Means ± Standard Error. Columns with different XY superscript indicate *p* < 0.0125 (Tukey-Kramer). Survey respondents were not required to answer every question, so n might not total 641.

**Table 4:** Correlations between self-reported number of MET hours per week during 18-month period of interest and BMD Z-score at each site N = 509. Correlations between body composition (in kg) and BMD Z-score at each site. (N = 631).

		Neck Z-Score		Troch Z-Sscore		Total Hip Z-Score		L1-L4 Z-Score	
MET Hours Category (Hours/Week)	Mean ± SD	R	*p*-Value	R	*p*-Value	R	*p*-Value	R	*p*-Value
Total MET Hours	90.7 ± 76.6	0.056	0.2218	0.057	0.2088	0.051	0.2587	0.004	0.9326
Moderate Activity (3–5.9 MET equivalents)	11.3 ± 9.3	0.021	0.6369	−0.005	0.9056	0.004	0.9363	0.033	0.457
Vigorous Activity (6–10.9 MET equivalents)	3.4 ± 4.1	0.095	0.0335	0.117	0.008 *	0.098	0.028	0.068	0.1271
Very Vigorous Activity (11–14.9 MET equivalents)	1.0 ± 2.1	0.074	0.0966	0.121	0.007 *	0.108	0.016	0.071	0.1118
Extremely Vigorous Activity (15–18 MET equivalents)	0.3 ± 1.1	0.010	0.8217	0.039	0.3856	0.032	0.4712	0.024	0.5823
Body Composition (kg)									
Total Bone Mass	2.4 ± 0.3	0.644	<0.0001 **	0.657	<0.0001 **	0.543	<0.0001 **	0.469	<0.0001 **
Total Fat Mass	20.9 ± 8.6	0.215	<0.0001 **	0.273	0.0001 **	0.225	<0.0001 **	0.098	0.027
Total Lean Mass	40.9 ± 5.2	0.437	<0.0001 **	0.468	<0.0001 **	0.413	<0.0001 **	0.134	0.002 **
Total Fat Free Mass	43.4 ± 5.4	0.615	0.025	0.485	<0.0001 **	0.423	<0.0001 **	0.153	0.008
Total Mass	64.3 ± 12.1	0.366	<0.0001 **	0.361	<0.0001 **	0.582	0.037	0.011	0.8459
VAT	0.2 ± 0.3	0.197	0.006	0.168	0.003	0.201	<0.0001 **	0.067	0.1318

* Statistically significant at *p* < 0.0125. ** Statistically significant at *p* < 0.005. Survey respondents were not required to answer every question, so n might not total 641.

## Data Availability

Data available upon request.
